# Novel Splicing Variants in the *ARR3* Gene Cause the Female-Limited Early-Onset High Myopia

**DOI:** 10.1167/iovs.65.3.32

**Published:** 2024-03-22

**Authors:** Jianing Niu, Weili Zhu, Xiaoying Jin, Xiaoming Teng, Junyu Zhang

**Affiliations:** 1 Reproductive Medicine Center, Shanghai First Maternity and Infant Hospital, School of Medicine, Tongji University, Shanghai, China; 2 Department of Obstetrics and Gynecology, Jiaxing Maternity and Child Health Care Hospital, College of Medicine, Jiaxing University, Jiaxing, China; 3 International Peace Maternity and Child Health Hospital, School of Medicine, Shanghai Jiao Tong University, Shanghai, China

**Keywords:** early-onset high myopia, X-linked inheritance, *ARR3*, splicing variants, minigene analysis, ClinGen gene curation

## Abstract

**Purpose:**

Variants in the *ARR3* gene have been linked to early-onset high myopia (eoHM) with a unique X-linked female-limited inheritance. However, the clinical validity of this gene–disease association has not been systematically evaluated.

**Methods:**

We identified two Chinese families with novel *ARR3* splicing variants associated with eoHM. Minigene constructs were generated to assess the effects of the variants on splicing. We integrated previous evidence to curate the clinical validity of *ARR3* and eoHM using the ClinGen framework.

**Results:**

The variants c.39+1G>A and c.100+4A>G were identified in the two families. Minigene analysis showed both variants resulted in abnormal splicing and introduction of premature termination codons. Based on genetic and experimental evidence, the *ARR3*–eoHM relationship was classified as “definitive.”

**Conclusions:**

Our study identified two novel splicing variants of the *ARR3* gene linked to eoHM and confirmed their functional validity via minigene assay. This research expanded the mutational spectrum of *ARR3* and confirmed the minigene assay technique as an effective tool for understanding variant effects on splicing mechanisms.

High myopia is a prevalent ocular disorder affecting a substantial portion of the global population.^1^ Early-onset high myopia (eoHM) is a rare condition characterized by severe nearsightedness that manifests in early childhood and has significant implications for vision and eye health. Individuals with eoHM face an elevated risk of retinal detachment, glaucoma, and myopic macular degeneration.^2^ eoHM is diagnosed based on refractive errors of −6 diopters or higher and onset before the age of 7 years, and it is associated with an increased likelihood of visual impairment and blindness.^3^ While environmental factors such as excessive reading or computer use and lack of outdoor activities can contribute to myopia development, genetic factors are predominantly regarded as the primary drivers of eoHM.^4^^,^^5^

Variants in the *ARR3* gene have been linked to X-linked female-limited eoHM, which is a rare disorder predominantly affecting females and exhibiting a female-limited inheritance pattern.^2^^,^^4^^–^^7^ Similar inheritance patterns are observed in conditions like epilepsy and cognitive impairment caused by *PCDH19* gene variants.^8^ *ARR3* is a 388–amino acid protein belonging to the arrestin family, crucial for regulating G protein–coupled receptor (GPCR) signaling and trafficking, impacting processes like vision and neurotransmission.^9^^–^^11^ It is expressed in the retina and pineal gland, desensitizing and internalizing light-activated rhodopsin to maintain retinal sensitivity.^12^^–^^14^ Its absence in zebrafish has been shown to reduce contrast sensitivity in cone-dominated vision.^13^ ARR4^–^^/^^–^ mice (*ARR3* ortholog) modulate high acuity vision and signaling pathways independently of ARR1 coexpression.^15^

In this study, we report two unrelated Chinese families with novel splicing variants in the *ARR3* gene. The pathogenicity of these variants was confirmed through RNA analyses. Furthermore, we conducted a comprehensive evaluation of the clinical relevance of *ARR3* and eoHM, categorizing them as “definitive.” Our findings contribute to the understanding of the genetic basis of eoHM and highlight the clinical significance of *ARR3*.

## Materials and Methods

### Patients and Ethical Approval

Two families with individuals affected by high myopia were recruited from the Reproductive Genetics Clinic at the International Peace Maternity & Child Health Hospital (IPMCH), Shanghai Jiao Tong University School of Medicine. Peripheral blood samples were collected from the participants, and comprehensive ophthalmologic examinations were conducted to confirm the diagnosis of myopia. Written informed consent was obtained from all participants. The study was approved by the Ethics Committee of IPMCH with approval number GKLW 2019-70.

### Ophthalmologic Phenotype Detection

Patients diagnosed with high myopia were defined as having a refractive error less than or equal to −6 diopters and axial length less than or equal to 26 mm. Detailed ophthalmologic examinations, including measurements of spherical equivalents, intraocular pressures, axial length, and fundus appearance, were performed for the individuals involved.

### Whole-Exome Sequencing and Sequencing Data Analysis

Genomic DNA was extracted from peripheral blood lymphocytes of specific family members (II2 in family I and III2 in family II). Subsequently, the DNA samples underwent sonication to break them into fragments of approximately 200 to 300 base pairs in length. Following this, Illumina paired-end sequencing libraries were prepared for whole-exome sequencing (WES) using the Agilent (Santa Clara, CA, USA) SureSelect Human All Exon V6 kit. The WES was performed on an Illumina (San Diego, CA, USA) HiSeq2500 analyzer. Clean sequencing reads were aligned to the human reference genome GRCh37/hg19 using the Burrows–Wheeler Aligner (BWA; v.0.7.12). Variant calling was carried out using the Genome Analysis Toolkit (GATK; v.3.8). The obtained variants were filtered and prioritized using Flash Analysis (https://fa.shanyint.com/). Additionally, Sanger sequencing was employed to validate the variants identified in the proband family through WES, confirming the genetic pattern and the existence of the variants. Variant classification was conducted in accordance with the American college of medical genetics and genomics (ACMG)/association for molecular pathology (AMP) guidelines and ClinGen specifications. The pathogenic criterion is weighted as very strong (PVS), strong (PS), moderate (PM), or supporting (PP).^16^^,^^17^

### Minigene Constructs and RNA Splicing Analysis

The DNA sequences containing the c.39+1G>A and c.100+4A>G variants, as well as the wild-type *ARR3* gene, were isolated from the human genome DNA. Subsequently, two different sets of primers were designed for DNA fragment amplification. Primers used for amplifying genomic regions covering exons 2 to 5 and exons 3 to 5 of the *ARR3* gene are listed in Supplementary Table S1.

The wild-type and mutant ARR3 amplicons were inserted into the pEGFP-C1 vectors. The recombinant minigene constructs with c.39+1G>A and c.100+4A>G were transfected into 293T/Hela cells or 293T/MCF-7 cells, respectively.

After 48 hours of transfection, total RNA was extracted using TRIzol reagent (TaKaRa, Shiga, Japan) and reverse transcribed to cDNA using HifairTM First Strand cDNA Synthesis SuperMix for qPCR (YEASEN, Shanghai, China). RT-PCR was conducted using primers listed in Supplementary Table S2.

The PCR products were analyzed by 1% to 3% agarose gel electrophoresis. All qPCR experiments were conducted using PrimerSTAR MAX DNA polymerase (TaKaRa) and analyzed using the QuantStudio 3 and 5 systems (Applied Biosystems, Waltham, MA, USA). Sanger sequencing were performed to analyze the splicing patterns of the minigenes.

### Curating Clinical Validity of *ARR3* and eoHM

The ClinGen gene curation framework was employed to evaluate the clinical validity between *ARR3* and eoHM. The disease entity and mode of inheritance associated with eoHM were established. A comprehensive search using the keyword “*ARR3*” in OMIM and PubMed was conducted to identify relevant primary literature. Original research articles providing details on the gene–disease relationship were gathered, and each piece of genetic and experimental evidence was curated and scored. The scoring system set a maximum of 12 points for genetic evidence and 6 points for experimental evidence.^18^ The strength of clinical validity was categorized as definitive (12–18 and replicated over time), strong (12–18), moderate (7–11), limited (0.1–6), no known disease relationship (0), or disputed (0, high population frequency).^18^

## Results

### Clinical Characteristics of Two Chinese Families With eoHM

#### Family I

**Figure 1.**
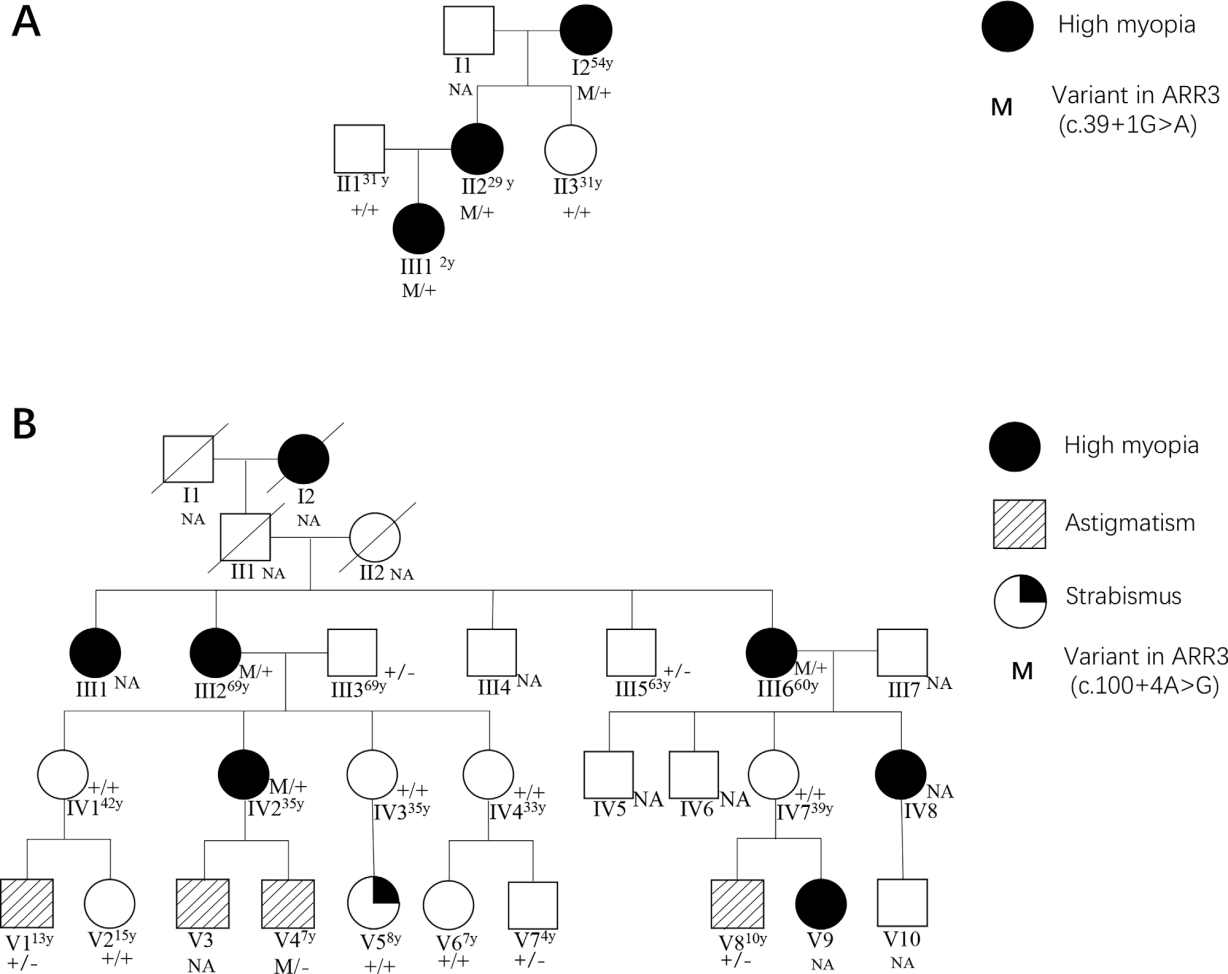
(**A**) Pedigree of family 1. II2 is the proband. (**B**) Pedigree of family 2. IV3 is the proband. Individuals subjected to Sanger sequencing are indicated as follows: “M/+” denotes mutant-type female heterozygote, “+/+” represents wild-type female homozygote, “M/−” signifies mutant-type male hemizygote, and “+/−” indicates wild-type male hemizygote. Individuals who did not undergo Sanger sequencing are labeled as NA. The superscript numerals affixed to lineage members denote the age at which the patients underwent Sanger sequencing or ophthalmic examination.

**Figure 2.**
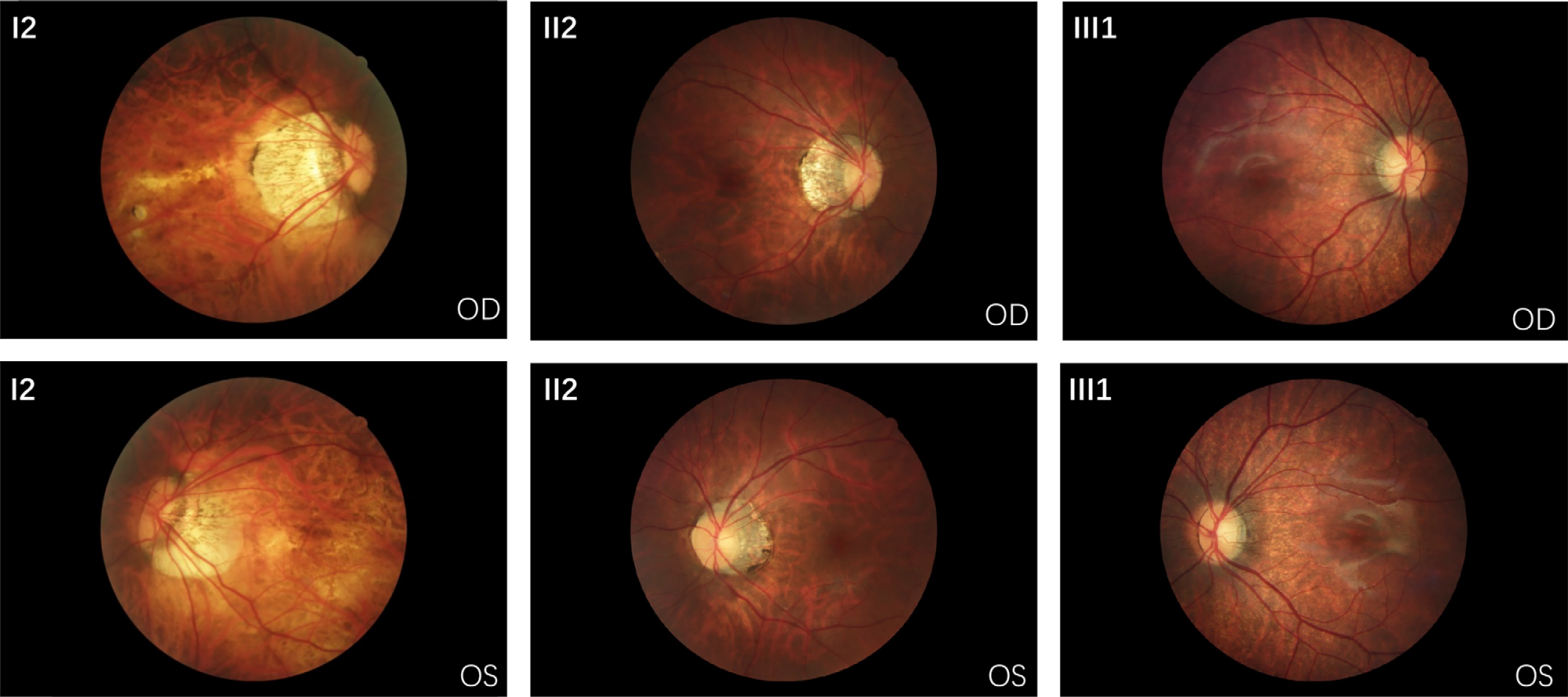
Fundus photographs of affected individuals in family I with heterozygous variant c.39+1G>A. These three patients all exhibit characteristic tiger-stripe fundus patterns associated with high myopia. The entire fundus appears dark gray due to diffuse choroidal atrophy, with a decrease or absence of choroid and intermediate-sized blood vessels. This results in the display of an orange-red large vessel layer, creating a tiger-stripe appearance. Patient I2 (54 years old) exhibits extensive retinal atrophy, an enlarged crescent atrophy, and even involvement of the peripapillary scleral region. The optic disc is significantly tilted, accompanied by severe choroidal vascular atrophy. The proband, patient II2 (29 years old), demonstrates a crescent-shaped choroidal atrophy around the optic disc, accompanied by disc tilting, forming a distinct vertical elliptical shape. Patient III1 (2 years old) shows the mildest presentation with no crescent atrophy, and the optic disc is in its normal position. OD, right eye; OS, left eye.

In family I, the proband (II2) presented with myopia, while her mother (I2) exhibited highly myopic conditions, and the proband's daughter (III1) was also diagnosed with high myopia. The proband sought consultation regarding the potential risk of her next pregnancy resulting in a highly myopic child. Notably, eoHM was observed in all female family members carrying the *ARR3* variant, including the young individual III1, who exhibited a high degree of myopia at −7.25/−7.75 diopters with axial length measuring 24.62/24.60 mm (Table 1, Fig. 1A). All family members exhibited distinct tigroid fundus changes (Fig. 2). Intraocular pressures measured in the proband and her mother's eyes were 12.1/11.0 mm Hg.

**Table 1.** Characteristics of Individuals in Families I and II

Individual	Age, Y	Gender	Variant in *ARR3*	Affected Status	Age on Set, Y	SER (Diopters)	AL (mm)	Complications
Family I			c.39+1G>A					
I1	NA	M	NA	U				
I2	54	F	M/+	A	<7	−14.75/−14.25	28.39/28.78	
II1	31	M	+/−	U				
II2	29	F	M/+	A	5	−3.25/−3.25	28.11/27.77	
II3	31	F	+/+	U				
III1	2	F	M/+	A	2	−7.25/−7.75	24.62/24.60	
Family II			c.100+4A>G					
I1	NA	M	NA	U				
I2	NA	F	NA	A	NA			
II1	NA	M	NA	U				
II2	NA	F	NA	U				
III1	NA	F	NA	A	<7			
III2	69	F	M/+	A	<7	−14.55/−14.75	28.46/28.73	
III3	69	M	+/−	U				
III4	NA	M	NA	U				
III5	63	M	+/−	U				
III6	60	F	M/+	A	5–6	−14.50/−14.70	28.40/28.75	
III7	NA	M	NA	U				
IV1	42	F	+/+	U				
IV2	35	F	M/+	A	5	−5.25/−5.25	28.39/28.31	
IV3	35	F	+/+	U				
IV4	33	F	+/+	U				
IV5	NA	M	NA	U				
IV6	NA	M	NA	U				
IV7	39	F	+/+	U				
IV8	NA	F	NA	A	4–6			
V1	13	M	+/−	U				Astigmatism
V2	15	F	+/+	U				
V3	NA	M	NA	U				
V4	7	M	M/−	U				Astigmatism
V5	8	F	+/+	U				Strabismus
V6	7	F	+/+	U				
V7	4	M	+/−	U				
V8	10	M	+/−	U				Astigmatism
V9	NA	F	NA	A	11			
V10	NA	M	NA	U				

A, affected; AL, axial length; F, female; M, male; NA, not available; SER, spherical equivalent refraction; U, unaffected.

#### Family II

In family II, the consultand (IV3) sought consultation due to her daughter's strabismus. Multiple highly myopic female patients were observed in her family. She inquired about the risk of having a highly myopic child in her next pregnancy. Medical records and family history were reviewed. The pedigree spanned five generations, and all seven affected patients (I2, III1, III2, III6, IV2, IV8, and IV9) were females with eoHM, suggesting a female-limited inheritance pattern. In addition to high myopia, some family members also exhibited mild astigmatism (V1, V3, V4, and V8) and strabismus (V5) (Table 1, Fig. 1B).

### Identification of Two Novel *ARR3* Splicing Variants by WES

WES was conducted using peripheral blood samples from the proband (II2) in family I and the consultand's mother (III2) in family 2. After applying data prioritization filters, a heterozygous novel canonical splicing variant, *ARR3* NM_004312.3: c.39+1G>A, was identified in the proband (II2) in family I. Additionally, a heterozygous novel noncanonical splicing variant, *ARR3* NM_004312.3:c.100+4A>G, was found in the consultand's mother (III2) in family II.

To validate these variants, Sanger sequencing was performed on PCR amplicons containing the identified variants, using available samples. Consistent with the WES results, the c.39+1G>A variant was detected in all patients (I2, II2, and III3) from family I. The c.100+4A>G variant was identified in three patients with high myopia (III2, III6, and IV2) and a male patient with mild astigmatism (V4).

The two variants identified in this study are novel and not present in gnomAD. Importantly, both variants are located at or near the canonical splice site, which typically leads to exon skipping.^16^ Furthermore, an analysis using SpliceAI (https://spliceailookup.broadinstitute.org/) indicates that both variants are likely to impact splicing. ∆scores, varying from 0 to 1, indicate the likelihood of a variant impacting splicing within a ±500-bp window, with detailed analysis for 0.2 (high recall), 0.5 (recommended), and 0.8 (high precision) thresholds. Specifically, the SpliceAI ∆score for *ARR3* NM_004312.3:c.39+1G>A is 0.8, and the SpliceAI ∆score for *ARR3* NM_004312.3:c.100+4A>G is 0.73. To confirm the splicing effects of these variants, minigene analyses were performed.

### The c.39+1G>A Variant Resulted in Aberrant mRNA Partially Retained Intron 3

**Figure 3.**
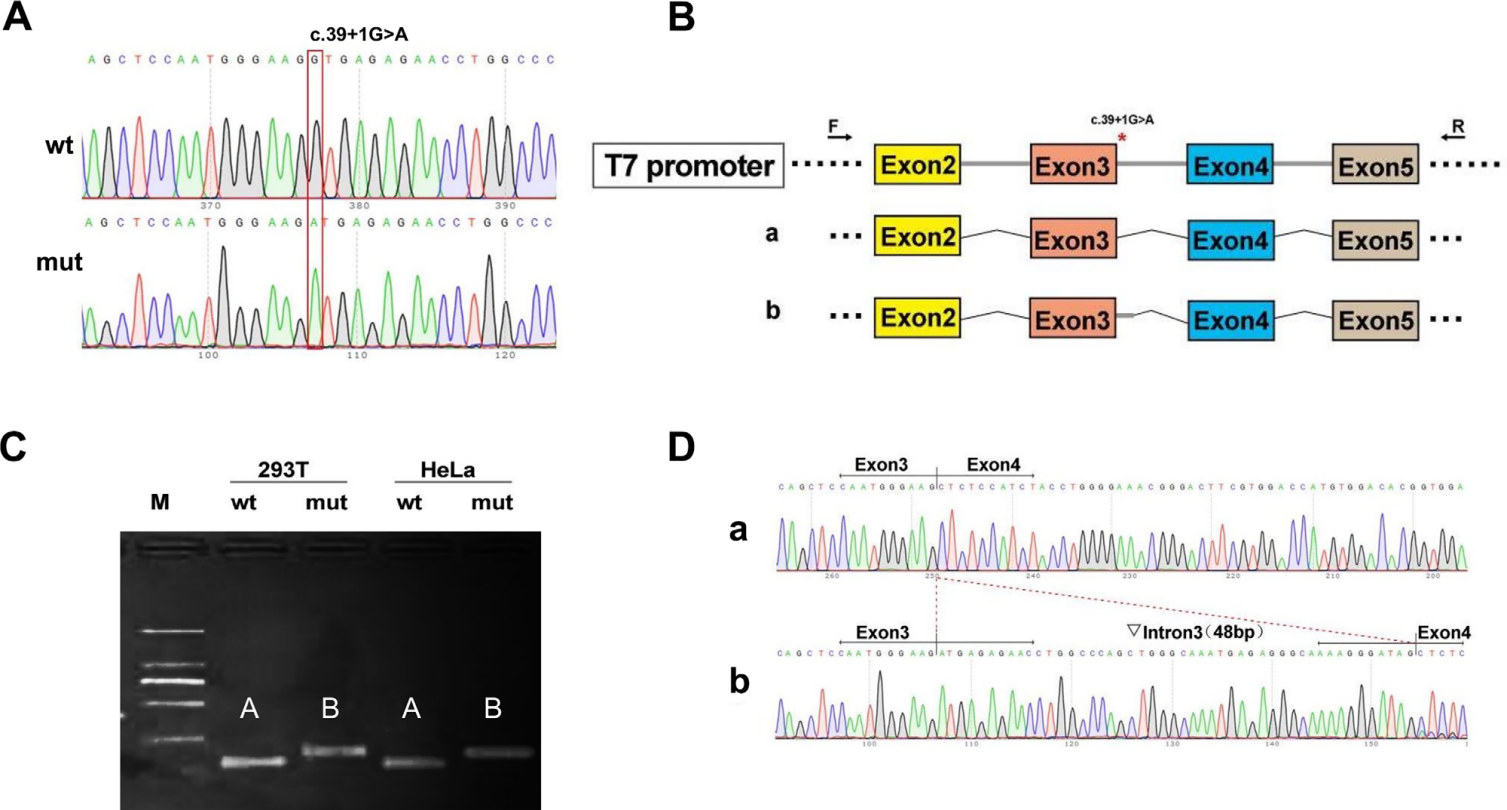
Transcription analysis results of c.39+1G>A. (**A**) Sequencing diagram of minigene construct, wild-type (wt) at the *top* and mutant (mut) at the *bottom*. (**B**) Minigene construction strategy and shearing diagram. (**C**) running gel of RT-PCR transcription analysis; the presence of differential bands in HeLa and 293T cells is labeled as A and B, respectively. (**D**) Sequencing results corresponding to the sheared bands. *Red* *asterisk* indicates variant location.

RT-PCR analysis showed that in 293T and HeLa cells, the wild-type construct produced a band of expected size, designated as band A, while the mutant construct produced a larger band compared to the wild type, referred to as band B (Fig. 3C). Both bands were confirmed by Sanger sequencing.

Sequencing results revealed that band A corresponded to a normal splicing pattern, with the following exon composition: exon 2 – exon 3 – exon 4 – exon 5. In contrast, band B exhibited an aberrant splicing event characterized by the retention of a 48-bp segment of the adjacent intron, leading to the following splicing pattern: exon 2 – exon 3 – ▽intron 3 (48 bp) – exon 4 – exon 5 (r.39_40insaugagagaaccuggcccagcugggcaaaugagagggcaaaagggauag). The aberrant mRNA resulting from the c.39+1G>A variant was predicted to produce a truncated protein (p.Leu14Metfs*16) (Fig. 3D). Consequently, the *ARR3* NM_004312.3: c.39+1G>A variant was classified as “pathogenic” based upon the following ACMG/AMP/ClinGen criteria: PVS1, PM2_Supporting, PP1.

### The c.100+4A>G Variant Resulted in Aberrant mRNA Partially Retained Intron 4

**Figure 4.**
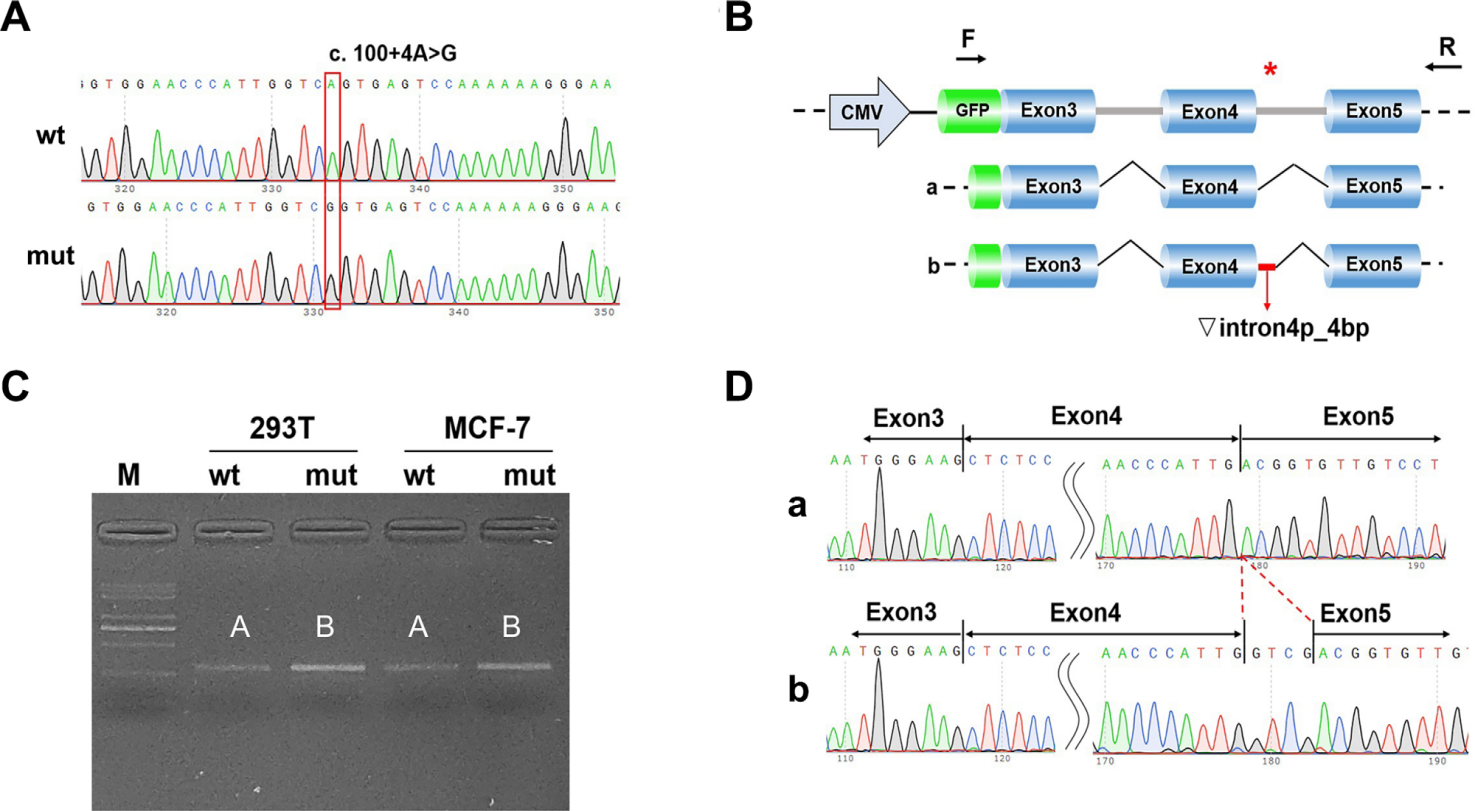
Transcription analysis results of c.100+4A>G. (**A**) Sequencing diagram of minigene construct, wild-type (wt) at the *top* and mutant (mut) at the *bottom*. (**B**) Minigene construction strategy and shearing diagram. (**C**) Running gel of RT-PCR transcription analysis; the presence of differential bands in HeLa and 293T cells is labeled as A and B, respectively. (**D**) Sequencing results corresponding to the sheared bands. *Red* *asterisk* indicates variant location.

The RT-PCR analysis revealed a band of expected size (296 bp), referred to as band A, in cells transfected with the wild-type construct (Fig. 4C). In the same time, cells transfected with the mutant construct showed a band, referred to as band B, of similar size to the wild type (Fig. 4C). Bands A and B were subjected to sequencing.

Sequencing results demonstrated that band A represented the normal splicing pattern, with exon 3 – exon 4 – exon 5. On the other hand, band B exhibited aberrant splicing, retaining a 4-bp segment from the adjacent intron, resulting in the following splicing pattern: exon 3 – exon 4 – ▽intron 4 (4 bp) – exon 5 (r.100_101insgucg). The abnormal mRNA produced by the c.100+4A>G variant was predicted to generate a truncated protein (p.Asp34Glyfs*16) (Fig. 4D). Consequently, the *ARR3* NM_004312.3:c.100+4A>G variant was classified as “pathogenic” based upon the following ACMG/AMP/ClinGen criteria: PVS1, PM2_Supporting, PP1.

### Case Summary and Clinical Validity Curation of *ARR3*/eoHM

The inheritance pattern of *ARR3* gene variants associated with eoHM has been proposed as X-linked with limited expression in females. We conducted a comprehensive review of relevant publications in PubMed, encompassing 36 families and 32 variants in the *ARR3* gene from both the literature and the two eoHM families included in this study (Supplementary Table S3). Additionally, we considered two animal studies that provided experimental evidence (Supplementary Table S4). Utilizing ClinGen's gene clinical validity curation process, we gathered and assessed all available genetic and experimental evidence related to *ARR3* and eoHM, as detailed in Table 2, compiled according to ClinGen’s standard operating procedure.^18^ With 12 points for genetic evidence and 6 for experimental evidence, totaling 18, and over 3 years since the original publication with >2 subsequent publications, we categorized the relationship between *ARR3* and eoHM as “definitive.”

**Table 2.** Genetic and Experimental Evidence Summary Matrix

		Guidelines				Points	
Evidence Type	Case Information Type	Default (per Variant)	Range (per Variant)	Variant Count	Proband Count	Total (Max)	Counted
Genetic evidence							
Case-level data							
Variant evidence							
Autosomal dominant or X-linked disorder	Predicted or proven null variant	1.5	0–3	21	25	32.6 (12)	12
	Other variant type	0.1	0–1.5	11	11		
Autosomal recessive disease	Predicted or proven null variant	1.5	0–3				
	Other variant type	0.1	0–1.5				
Segregation evidence			Range	Summed LOD	Family Count	2.1 (3)	2.1
	Candidate gene sequencing		0–3	7.8	2		
	Exome/genome or all genes sequenced in linkage region			5.7	2		
	Total summed LOD score			13.5			
		**Guidelines**				**Points**	
**Case-Control Study Type**	**Case-Control Quality Criteria**	**Points/Study**		**Count**		**Total**	**Counted**

Case-control data							
Single variant analysis	1. Variant detection methodology 2. Power 3. Bias and confounding 4. Statistical significance	0–6					
Aggregate variant analysis		0–6					
	Total genetic evidence points (maximum 12)						12
		**Guidelines**				**Points**	
**Evidence Category**	**Evidence Type**	**Default**	**Range**	**Max**	**Count**	**Total**	**Counted**

Experimental evidence							
Function	Biochemical function	0.5	0–2	2	2	1	2
	Protein interaction	0.5	0–2		1	0.5	
	Expression	0.5	0–2		2	1	
	Functional alteration	Patient cells	1	0–2	2		1
	Nonpatient cells	0.5	0–1		2	1	
Models	Nonhuman model organism	2	0–4	4	2	4	4
	Cell culture model	1	0–2				
Rescue	Rescue in human	2	0–4				
	Rescue in nonhuman model organism	2	0–4				
	Rescue in cell culture model	1	0–2				
	Rescue in patient cells	1	0–2				
Total experimental evidence points (maximum 6)							6

LOD, logarithm of the odds.

## Discussion

In this study, we identified two novel heterozygous splicing variants in the *ARR3* gene, c.39+1G>A and c.100+4A>G, segregating with eoHM in two Chinese families. Using minigene splicing assays, we demonstrated that both variants resulted in aberrant splicing through retention of intronic segments, introducing premature termination codons likely leading to truncated, nonfunctional proteins. Our findings expand the mutational spectrum of *ARR3* variants associated with eoHM and provide functional evidence supporting their pathogenicity.

Certain genes are reported to affect the development of eoHM. *COL2A1* gene variations can result in syndromic eoHM, associated with eye connective tissue, vitreous degeneration, deafness, joint problems, and facial abnormalities due to the production of type II collagen.^19^^–^^22^ Other genes such as *LEPREL1*, *OPN1LW*, *OPN1MW*, and *ZNF644* are linked to nonsyndromic eoHM. *LEPREL1* codes for an enzyme involved in collagen modification.^23^^,^^24^ Variants here can result in severe nonsyndromic eoHM and early-onset cataracts. *OPN1LW* and *OPN1MW* encode opsin proteins in the retina. Variants in these genes can lead to high myopia and affect color perception.^25^^,^^26^ *ZNF644*, a gene related to eye development, can also be associated with high myopia.^27^^,^^28^ The exact genes and mechanisms causing eoHM are not fully understood and require further research.

*ARR3* exhibits unique female-limited X-linked heritability in eoHM.^2^^,^^6^^,^^7^ However, the extent of the relationship between *ARR3* and eoHM has not been systematically determined through ClinGen gene clinical validity curation. By integrating our findings with previous evidence, we systematically evaluated the clinical validity of the *ARR3*–eoHM gene–disease relationship using the ClinGen framework. The wealth of genetic evidence from linkage studies, case reports, and animal models enabled classification of this association as “definitive.” This designation provides clinicians with reliable information to facilitate genetic diagnosis and counseling for individuals and families with eoHM.

The female-specific expression pattern can be explained by random X chromosome inactivation and the resultant tissue mosaicism in affected females. During embryonic development in females, one of the two X chromosomes is randomly and permanently inactivated in each cell to achieve dosage compensation with males. This stochastic process generates a mixture of cell lineages expressing either the wild-type or mutant *ARR3* allele. In contrast, hemizygous males have a homogeneous cell population expressing the single X chromosome copy. The variable ratio of wild-type to mutant cells due to random X inactivation accounts for the intrafamilial phenotypic heterogeneity observed between affected sisters sharing the same variant. A recent study found that a proband with 81.3% of cells expressing the mutant *ARR3* allele had milder eoHM than her sister, who had 56.12% mutant expression.^29^ This observation suggested a correlation between the severity of the phenotype and the ratio of the cell population. This mosaic expression pattern facilitates manifestation of X-linked conditions in heterozygous females, indicating phenotypic variability within families harboring identical mutations. For instance, in family I, the proband (II2) was diagnosed with myopia, whereas her mother and daughter presented with high myopia.

The newly identified variant at position c.100+4 is not a conventional donor/acceptor ±1,2 splicing variant.^30^ Minigene analysis revealed that the variant disrupts normal mRNA splicing, resulting in the retention of a 4-bp segment on the right side of intron 4. This observation suggests the presence of a potential donor splice site at position c.100+5, with the variant c.100+4A>G activating this site for recognition by the splicing complex. The resulting 4-bp insertion generates a premature stop codon, leading to premature protein termination. Variations in splice sites often disrupt the recognition of exons, leading to exon skipping during the splicing process.^31^^,^^32^ In our study, two variants from different families were located near splice sites, suggesting their potential interference with normal RNA splicing. However, it is important to note that the *ARR3* gene is specifically expressed in the retina, and *ARR3* mRNA is not detectable in peripheral blood.^33^ To determine whether these two variants indeed cause exon skipping and subsequent loss of amino acids, mutant DNA templates were extracted from both families for minigene transcription. The minigene analysis precisely demonstrated the alteration of this variant on RNA splicing sites and amino acid truncation, without resulting in the anticipated exon skipping. Therefore, experimental verification of variants remains crucial. We recommend employing minigene tests as a reliable method to confirm the impact of variants on RNA splicing and their effects on protein products.

In family II, we observed a peculiar phenomenon within the fifth generation, where various visual abnormalities were present, including high myopia, astigmatism, and strabismus. Among the affected individuals, V1, V3, V4, and V8 exhibited astigmatism, while V5 displayed strabismus. These visual abnormalities could be attributed to other pathogenic genes or possibly influenced by environmental factors. V4 carried an *ARR3* variant (c.100+4A>G) but did not exhibit eoHM, which is consistent with a genetic pattern limited to females. In the case of individual V9, given that the maternal lineage (individual IV7) is negative for the *ARR3* variant, the probability of V9 inheriting this variant is markedly reduced. However, the lack of Sanger sequencing analysis for both V9 and V9’s paternal lineage prevents definitive exclusion of V9 as a carrier of the variant.

In conclusion, our study expands the allelic spectrum of *ARR3* variants underlying eoHM pathology and provides functional evidence supporting their pathogenic impact. We demonstrate the utility of minigene splicing assays to evaluate effects of variants at the mRNA level. The intriguing inheritance patterns and genotype–phenotype correlations in this X-linked form of high myopia warrant continued investigation. In this study, we curate the clinical validity of *ARR3* and eoHM as a “definitive,” facilitating genetic diagnosis and counseling for this condition. Elucidating the precise genetic mechanisms mediating *ARR3*-associated eoHM may reveal therapeutic targets to guide management.

## Supplementary Material

Supplement 1
