# Supplementary material for: Novel Splicing Variants in the ARR3 Gene Cause the Female-Limited Early-Onset High Myopia
Source: Invest Ophthalmol Vis Sci. 2024 Mar 22;65(3):32. doi: 10.1167/iovs.65.3.32 (PMC10981162; doi:10.1167/iovs.65.3.32)
Supplement: Supplement 1 [file iovs-65-3-32_s001.pdf]

Supplementary materials

Table S1. Primer sequences designed for amplifying exons 2-5 and exons 3-5 of the *ARR3* gene

***ARR3*: c.39+1G>A**	
ARR3-692 Forward	CTCTCTCAGGGTTCTCAAGAGTT
ARR3-2175 Reverse	CACCACCAGTAATAATAGAGA
***ARR3*: c.100+4A>G**	
ARR3-1010 Forward	GCTCAACATCAACTATATAGCC
ARR3-2120 Reverse	CAGCACAAGTGTAAGTTTTGTG

Table S2. Primer sequences designed for minigene construction

***ARR3*: c.39+1G>A**	
Forward primer for EGFP-C1-*ARR3*-Kpn1	GCTTGGTACCATGTCCAAGTAAGAATTCCTTC
Reverse primer for pEGFP-C1-*ARR3*-BamH1	TAGTGGATCCCAACTTTCGACATTTTAAGTACTC
***ARR3*: c.100+4A>G**	
Forward primer for EGFP-C1-*ARR3*-KpnI	CGACGGTACCAAGGTGTTTAAGAAGACCAGCT
Reverse primer for EGFP-C1-*ARR3*-BamHI	CGGTGGATCCCAACTTTCGACATTTTAAGTAC

Table S3. Case-level data in families with *ARR3* (NM_004312.3) variant

No.	Variants in *ARR3*	Segregation counting	Null variant	Functional data	De Novo	Complications	Genotypes and Phenotypes of family members	PMID
F1	c.767+1G>A, p.?.	3	√				5 patients(5 females)<br>4 of the 15 individuals carried the variant(3 females and 1 male). Proband A had a nearsightedness of −8.0/−9.5 diopters, an AL of 26.75/26.09 mm. Proband B had a nearsightedness of −8.0/−8.75 diopters, an AL of 26.09/26.08 mm.	35001458
F2	c.214C>T, p.Arg72*	2				Myopic macular degeneration	8 patients<br>Proband A had a nearsightedness of −18.75/−16.00 diopters, an AL of 30.46/29.70 mm.Aunts D and C have high myopia. Nieces G and L have high myopia; niece G suffered Retinal detachment at age 18. Deceased grandma M and her deceased mother O and her deceased sister N all were affected by high myopia.	
F3	c.848delG, p.Gly283fs	1	√			Ocular migraines, recurrent nasotemporal vision loss. Posterior staphyloma (right eye)	5 patients<br>Proband A had a nearsightedness of −15.75/−11.00 diopters, an AL of 29.40/28.03 mm.Sister C has high myopia; her 1-year-old daughter (D) has progressing early onset high myopia. Half-sister E also suffered from high	

							myopia.<br>Grandma H was also been afflicted with high myopia	
F4	c.893C>A,p.Ala298Asp	17					15 patients	27829781
F5	c.298C>T,p.Arg100*	9	√				10 patients	
F6	c.239T>C,p.Leu80Pro	3					6 patients	
F7	c.214C>T, p.Arg72*	13	√			Fundus appearance: tesselated retina, incipient diffuse chorioretinal atrophy<br>peripapillary atrophy<br>OCT: Incipient atrophic sensory retina	14 patients<br>8 of family members were exposed to a more thorough examination, 6 of them were patients.<br>IV-1 had a nearsightedness of -8/-8 diopters, an AL of 26.34 / 26.24 mm,<br>IV-2 had a nearsightedness of -6/-4 diopters.<br>IV-6 had a nearsightedness of -23/-19 diopters, an AL of 30.12 / 29.81 mm,<br>IV-7 had a nearsightedness of −13/−9.5 diopters, an AL of 27.45 / 26.1 mm,<br>III-8 had a nearsightedness of −14/−7 diopters.<br>IV-10 had a nearsightedness of −12.5/−14.5 diopters, an AL of 27.02 / 26.971 mm,	33482870

F8	c.3G>A,p.Met1?	6	√			Astigmatism ranged from −0.25 D to −6.00 D (−2.63 D±1.32D). BCVA, ranged from 0.01 to 1.20	6 patients (5 females and 1 male) had refractive errors from −5.00 D to −28.75 D (−12.58D±4.83D)<br>8 of the 15 individuals carried the variant(6 females and 1 male)	36180177
F9	c.9-1G>A,p.?.	6	√				In a family of 3, the mother and son carried the variant and were both affected.	
F10	c.103G>A,p.Gly35Ser	0			√		In a family of 3, the daughter carried a denovo variant, and was affected.	
F11	c.139C>T,p.Arg47*	0	√		√		In a family of 4, one daughter carried a denovo variant, and was affected.	
F12	c.139C>T,p.Arg47*	1	√				In a couple with 4offspring, the mother and one daughter carried the variant and were affected.	
F13	c.146T>G,<br>p.Leu49Trp	1					In a family of 4, the mother and a daughter carried the variant, and were affected.	
F14	c.149T>C,p.Phe50Ser	1					In a family of 3, the mother and daughter carried the variant and were both affected.	
F15	c.232C>T,p.Gln78*	2	√				5 patients in a famliy of 12.<br>2 females and 1male carried the variant,2 females didn't carry the variant.	
F16	c.298C>T,p.Arg100*	0	√		√		In a family of 3, the daughter carried a denovo variant, and was affected	
F17	c.298C>T,p.Arg100*	0	√				In a family of 3, the father and daughter carried the variant, and the daughter was affected.	
F18	c.345G>C,p.Gln115Hi	0			√		In a family of 4, the father and a daughter	

	s						carried the variant, and the daughter was affected.	
F19	c.346-2A>T,p.?.	0					In a family of 3, the daughter carried a denovo variant, and was affected. The mother was affected too.	
F20	c.361C>A,p.Pro121Thr	0					In a three-generation family of 7， the granddaughter carried a denovo variant, and was affected. The grandmother was affected too.	
F21	c.386_389del,p.Gly129fs	1	√				In a family of 4, two daughters carried the same variant and were both affected.	
F22	c.499A>T,p.Lys167*	0	√				In a family with 3 children, the mother and one daughter were affected. The daughter carries the variant, but the mother's genotype is not available.	
F23	c.520G>T,p.p.Glu174*	0	√		√		In a family of three, the daughter carried a denovo variant, and was affected	
F24	c.520delG,p.Glu174fs	0	√				In a three-generation family of 8， the grandma, the mother and a grandson were affected. The grandson carried the variant, but the genotype of other individuals were not available.	
F25	c.707C>G,p.Thr236Arg	0					In a family of 3, the daughter carried the variant, and was affected.The genotype of the parents were not available.	
F26	c.757delC,p.Gln253fs	0	√				In a family of 4, the daughter carried the variant, and the genotype of the rest were not available. Both the daughter and the mother were affected.	

F27	c.844_845insT,p.Arg282fs	3	√				In a three-generation of 9, the grandma, two her daugther and one grand daugher were carried the variant, and all of them were affected.	
F28	c.928G>T,p.Glu310*	1	√				In a family of 3, the mother and daughter carried the variant and were both affected.	
F29	c.929_930del,p.Glu310*	0	√		√		In a family of 4, the father and a daughter carried the variant, and the daughter was affected.	
F30	c.963_964del,p.Arg321*	1	√				In a three-generation of 7, the grandma, the daughter and the grand daughter were affected. The daughter and the grand daughter carried the variant, but the genotype of other individuals were not available.	
F31	c.963_964del,p.Arg321fs	0	√		√		In a family of 3, the daughter carried a denovo variant, and was affected.	
F32	c.963_964del,p.Arg321fs	0	√				In a three-generation family of 14, the grandmother, a daughter and 2 grand daughter were affected. One of the grand daughter carried the variant and the other was not available.	
F33	c.1041-2A>G,p.?.	0			√		In a family of 3, the daughter carried a denovo variant, and was affected	
F34	c.666delC,p. Asn222Lysfs*	2	√				4patients<br>The proband(III3) had a refractive error of 7.75/-9.50 diopters and an AL of 24.41/24.57 mm<br>The cousin (III4) had a refractive error of −4.50/−5.00 diopters and an AL of	36769483

							27.26/27.55 mm at 11 year-old. The aunt (II3) showed high myopia of -2.25/-1.75 diopters with ALs of 24.41/24.57 mm.	
F I	c.39+1G>A, p.Leu14Metfs*	2	√	√		tigroid appearance	3 patients<br>The proband(II2) had a refractive error of −14.75/−14.25 diopters and an AL of 28.39/28.78 mm The mother (I1) had a refractive error of −14.75/−14.25 diopters and an AL of 28.39/28.78 mm. The daughter (III1) showed high myopia of -7.25/-7.75 diopters with ALs of 24.62/24.60 mm at 2 years old . All affected female family members present typical tigroid fundus changes.	
F II	c.100+4A>G, p.Asp34Glyfs*	2	√	√			7 patients<br>I2, III1, III2, III6, IV2, IV8, and V9 had high myopia<br>V1, V3, V4, and V8 had astigmatism, while V5 had strabismus.	

Table S4. Experimental evidence in *ARR3* gene

Experiment	Function	Functional Alteration	Models	Rescue	PMID
Experiment1	Biochemical function<br>Protein interaction<br>Expression	Non-patient cells	Zebrafish model	No	21299656
Experiment2	Biochemical function<br>Expression	Non-patient cells	Mice model	No	26284544
